# Characteristics of Cognitive Abilities among Youths Practicing Football

**DOI:** 10.3390/ijerph18041371

**Published:** 2021-02-03

**Authors:** Wojciech Paśko, Maciej Śliż, Mariusz Paszkowski, Janusz Zieliński, Klementyna Polak, Maciej Huzarski, Krzysztof Przednowek

**Affiliations:** 1 Institute of Physical Culture Sciences, Medical College of Rzeszów University, Rzeszów University, 35-959 Rzeszów, Poland; msliz@ur.edu.pl (M.Ś.); jzielinski@ur.edu.pl (J.Z.); kpolak@ur.edu.pl (K.P.); mhuzarski@ur.edu.pl (M.H.); krzprz@ur.edu.pl (K.P.); 2 Football Lab Company, 05-092 Dziekanow Lesny, Poland; mariusz.paszkowski@footballlab.pl

**Keywords:** cognitive abilities, psychomotor abilities, youth sports, football players, reaction time, motor time, simple reaction time, complex reaction time, hand-eye coordination, spatial orientation

## Abstract

The aim of the study was to assess selected cognitive abilities depending on age, anthropometric parametres, physical fitness and technical skills in the group of young players training football. The study covered a group of 258 young players practicing football (age: 12.1± 2.03), who were divided into 5 age categories (8–9 years old, 10–11 years old, 12–13 years old, 14–15 years old, 16–17 years old). Selected cognitive abilities include: simple reaction time (SIRT), complex reaction time (CHORT), hand-eye coordination (HECOR) and spatial orientation (SPANT). Studies were performed using Test2Drive computer tests. In addition, the level of physical fitness was measured using: The standing long jump, 30 m sprint, 20 m shuttle run test (without and with the ball) and slalom (without and with the ball). The analysis showed a statistically significant relationship between age and cognitive abilities. There was also a statistically significant correlation between fitness tests and reaction time in individual cognitive tests. There were no statistically significant relationships between technical skills and cognitive abilities. The study confirms that age and physical fitness affect the level of cognitive abilities.

## 1. Introduction

Obtaining the best possible result in sport is associated with the acquisition of motor skills by players which directly affect the performance of movement i.e., speed, automation, precision and adaptability [1]. The motor skills include coordination skills of which one of the components is reaction speed [2]. The speed of reaction determines the speed of execution of a given movement which is a response to a given stimulus caused by a signal or a change in the situation [3]. There are many factors that influence the speed of reaction, including muscle arousal, type of stimulus, age, gender, left or right handedness, direct and peripheral vision, practice and mistakes, fatigue, distraction, stimulants, intelligence, stress, diseases, type of personality, etc. [4,5]. Reaction speed includes reaction time and motor time [6]. The reaction time is the time from the initiation of the stimulation in the receptor to the activation in the muscle. Research has shown that this factor is determined by individual characteristics and cannot be influenced by training [7,8]. The motor time, on the other hand, is the time from the stimulation of the muscle to the performance of a given motor task [9]. This motor ability is particularly influenced by the neuromuscular coordination of motor units, which we can shape through appropriate training. In sport, there is a simple reaction time when there is one stimulus and one response to it, e.g., a sprint start [10]. The second type is a complex reaction time, while for a given stimulus we have the option of choosing the answer, e.g., table tennis [11].

Many team sports, such as American and Australian football, rugby, ice and field hockey, basketball, handball or football are characterized by a constant change of intensity, physical contact with the opponent, and the presence of multiple complex motion activities during the game [12]. Sports players who have a direct contact with the opponent have to cope with a constant adaptation to the situation imposed by the opponent. A very important element for the player, despite physical effort, is to react to the changing environment in the shortest possible time in order to gain an advantage and score [13,14]. Sports disciplines at the highest level require the best psychomotor preparation of players because every detail can affect the final sports result [15,16]. There are many studies confirming that players practicing various sports disciplines are characterized by a better level of cognitive abilities than non-training people [17,18,19,20,21,22,23,24,25].

Football is considered the most popular sport discipline over the world [26]. It is a team game characterized by varying intensity and unpredictable situations that dynamically change during the game [27]. It is considered that responsiveness in football is one of the key abilities that determine the technical and tactical skills, regardless of the player’s position on the pitch [28]. Moreover, the response time may also be related to the risk of musculoskeletal injury [29,30]. During a study on professional football players, the correlation between the response time and speed achieved on a 20-m distance was checked [23]. Aksoy et al. [31] compared the response times of young football players, volleyball players and wrestlers where football players achieved the best time. The comparison of the reaction time between football players and non-training people was also analyzed by Ando et al. [32]. These studies also showed that football players achieved better reaction time.

The skills of young football players in different age categories were analysed by Figueiredo [33], showing that with age football skills and physical fitness are at a better level. The responsiveness of the upper and lower limbs to visual stimuli was measured among young players of Valencia Soccer Club and compared with the control group. The studies showed that young football players had better visual reaction time in both cases [34]. Young players were also subjected to tests to examine the impact of fatigue on responsiveness. It was also found that the reaction time gradually decreased with increasing the effort to about 75% $VO_{2}$, where then, it sharply increased [35]. Lemmink et al. [36] did not show any significant correlation between intermittent exercises performed by young football players and the time of multiple-choice reactions. Further research showed that professional players have a better complex reaction time which suggests that this may be a parametre used to identify talents among young football players [37]. The similar conclusions had Verburgh et al. [38] who compared boys participating in the talent development program at the youth academy of the Dutch Premier League and players from the lower leagues. Huijgen et al. [39] showed that cognitive abilities depend on the level of training of young footballers. The measurement of the response time was also performed on young players of the German 1st league who were divided into age categories U12, U13, U17 and U19. The study shows that with age reaction time improves [40]. While Penna et al. [41] showed that reaction speed did not depend on the time of birth in the group of young footballers under the age of 13. Hirose et al. [42] analyzed changes of the response time due to chronological and skeletal age in the group of football players between the age of 10 to 15 where reaction time improves with age. While Vänttinen et al. [43] examined an improvement of response time with age in young footballers group. A similar analysis was performed in the studies by Iida et al. [44] where changes in cognitive abilities were observed in the group of children between 6 to 12 years old. While Schumacher et al. [2], apart from reaction time, also analyzed the relationship between the level of concentration and the age of young football players. The relationship between the level of body fat and reaction time were also analyzed in young players training football, where there was no significant relationship [45].

In order to extend knowledge about the importance of responsiveness in youth football, the authors of the paper analyzed the structure of psychomotor skills. The aim of the study was to assess selected cognitive abilities depending on age, anthropometric parametres, physical fitness and technical skills in the group of young player training football.

## 2. Material and Method

### 2.1. Characteristics of the Study Group

The study group consisted of 258 boys training football between the age of 8 and 16 years (age: 12.1±2.03). Players were participants of football camps organized by the Football Lab, during which the tests were conducted. The study was conducted on young football players divided into 5 different age groups (Table 1), G1—8–9 years old, G2—10–11 years old, G3—12–13 years old, G4—14–15 years old, G5—16 years old. Each of these age categories has its own system of games and rules laid down by the Polish Football Association. The study was carried out on summer camps 2020. The scope and project research were assessed by the Ethics Committee of the University of Rzeszow/Poland (resolution 10/02/2020).

The parents and Football Lab gave written consent allowing the young players to take part in the study, while the researchers guaranteed that the collected data would be treated confidentially.

### 2.2. Measurement of Psychomotor Abilities

In this study, measurements included the assessments of the level of cognitive abilities [46] and special fitness tests in the group of young football players. The studies were divided into two days. During the first day young football players had the measurement consisted of four computer system tests which were used to measure the level of cognitive abilities.

The measurements were taken in a standing position so that the participants could perform certain tasks freely. Before the beginning of each test, the participant was informed about its principles and then the proper tests were carried out. In each type of test the stimuli appeared at different intervals. Computer tests of cognitive abilities consisted of:

(1) SIRT- was the first test to measure simple response time. The purpose of the measurement was to assess the rate of response and its stability. The participant was intended to move his finger to the blue field as quickly as possible. Each measurement began with the “START” field.
(2) CHORT- was the second test, choice reaction time test, which was designed to evaluate the speed and adequacy of the reaction in a complex situation. The participant was intended to respond to stimuli in the form of vertical or horizontal lines, while during the appearance of stimuli in the form of diagonal lines, there was no reaction and the finger was supposed to remain on the “START” field. During this test, the measurement also included incorrect answers.
(3) HECOR- the third measurement test was the hand-eye coordination test. The test evaluated time needed to analyze and initiate movement and its speed. The task for the participant was to move his finger to the blue field, which was equivalent to the red field and return to the “START” field as soon as possible.
(4) SPANT- the fourth measurement test was a two-dimensional spatial anticipation test. The test was based on an assessment of the ability to plan movement where spatial orientation is important. The participant was asked to select a crossing field of two red dots that were illuminated. The red points were placed horizontally at the top of the screen and vertically at the left and right sides of the screen. During this measurement, the incorrect answers were also taken into account.

On the second day, the measurements consisted of special performance tests, which were used to estimate the level of fitness. Before the fitness test all participants had a warm-up at a football camp. The mean ambient temperature during the research was 22 °C. It was sunny with no rain. The altitude of the pitch where the research was conducted was 210 m above sea level. All tests were carried out on the pitch in the same order: standing long jump, 30-m sprint, shuttle running with and without a ball, slalom run with and without a ball. After each of test taken football players had a break.

(1) Standing long jump (SLJ)– the distance of the jump was measured.
(2) 30-m sprint (S30)– the time was measured over a distance of 5 m, 10 m and 30 m. The player decided himself about the start of the race.
(3) Shuttle running with and without a ball (T1)– the player decided when to start and then run around the cones which were placed at 2 m, 4 m, 6 m, 8 m and ends at 10 m. After each cone, he returned to the starting point.
(4) Slalom run with and without a ball (T2)– the distance between the start line and the first cone was 2 m and the distance between each successive cone was 4 m. However, the distance between the last cone to the finish line was 2 m. The player decided when to start the race.

### 2.3. Statistical Methods

The normality of distributions was assessed by using the Shapiro-Wilk test. Most of the variables have a non-normal distribution therefore the non-parametric tests were used. Basic statistical measures i.e., arithmetic mean, standard deviation and coefficient of variability were calculated. Differences between age groups were evaluated by the non-parametric Kruskal Wallis test. The relationships between cognitive abilities, physical fitness and anthropometric parametres were analyzed using the Spearman correlation. Additionally, forward selection multiple regression models were designed to investigate in detail the analysed relationship. The fitting of regression models was assessed by leave-one-out-cross-validation (LOOCV). During the LOOCV RMSE and NRMSE errors were calculated which has the form:

(1) $$RMSE=\sqrt{\frac{1}{n}\sum_{i=1}^{n}{\left(y_{i}-{\hat{y}}_{-i}\right)}^{2}},\;NRMSE=\frac{RMSE}{\bar{y}}\cdot 100,$$

where: RMSE–root mean squared error, NRMSE–normalized root mean squared error, *n*–total number of data, ${\hat{y}}_{-i}$–the output of a model calculated after removing the pair $\left(x_{i},y_{i}\right)$, $\bar{y}$–arithmetic average of y from total number of data. The R software (R Foundation for Statistical Computing, Vienna, Austria) was used for the study’s statistical analysis [47].

## 3. Results

Table 2 shows the results of psychomotor tests for each age group. It was noticed that the differences in the results of individual tests are statistically significant (*p* < 0.05). Both for reaction time (RT) and motor time (MT), the youngest age group G1 obtained the longest time. In the SIRT test the shortest time was reached by the most numerous age group G3, where the RT was (371 ms) and the MT was (177 ms). In the CHORT test, the oldest group G5 achieved the shortest time for RT (711 ms), while the G3 age group had the fastest time in MT, the only one with a result below (200 ms). The best results with the highest number of correct answers was noted in the G4 age group where the correctness of the answers was 91.5% while G1 age group had only 69.6% of correct answers. In the HECOR test, G3 age group had the shortest MT (220.5 ms). In RT HECOR test, the G4 age group obtained the best result. In the last SPANT test for RT, the best result achieved the oldest age group G5 (637 ms), which also had the most correct answers (90%). The age group G4 (257 ms) noted the shortest MT SPANT. While in SPANT test G1 age group had the least correct answers, where the correctness of the answer was 51%.

The next stage of the research was to analyze the statistical significance between the differences in the results of individual age groups and psychomotor tests (Table 3). In case of differences between groups G1 and G3, statistical significance appeared in each test. Furthermore, statistical significance for the difference in results between groups G4 and G5 were only in MT SPANT. For the rest of tests, the differences are not statistically significant. For CHORT c.r and SPANT c.r, the statistical significance of the differences inresults does not occur only between the G4 and G5 groups.

Table 4 shows the results of fitness tests which were conducted on each age groups. With age, the results of fitness tests were better in each group.

**Figure 1:**
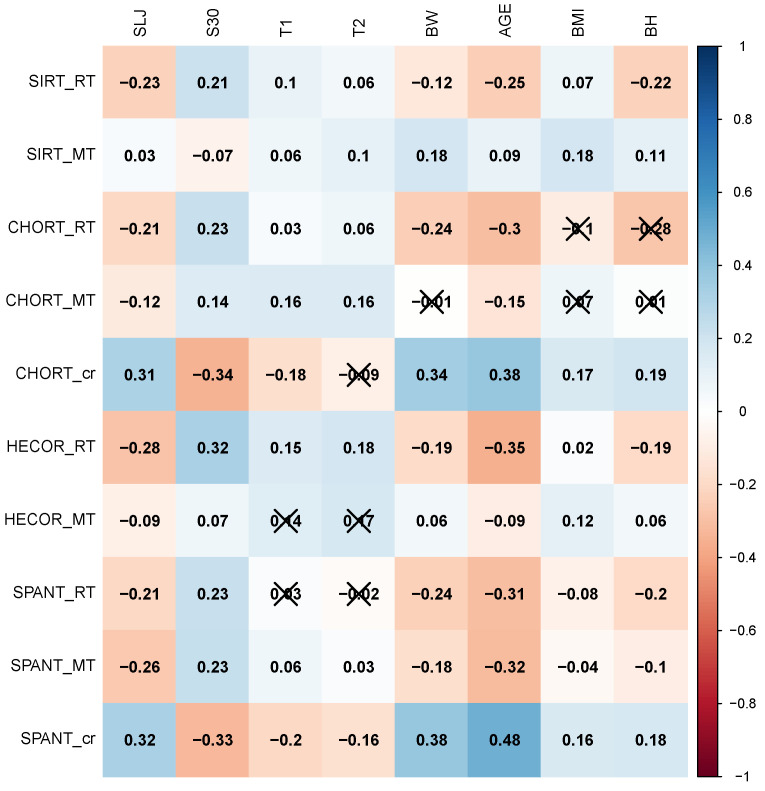
Correlations graph.

Figure 1 presents the correlation between cognitive abilities, anthropometric parametres and fitness tests. The vast majority of the tested compounds show statistical significance. The highest positive correlation can be observed between the number of correct answers in the SPANT test (SPANT_cr) and age (0.48). On the other hand, the highest negative correlation was between the RT HECOR psychomotor test and age (−0.32). RT SIRT low correlation occurred between the standing long jump (−0.23), 30 m sprint (0.21), age (−0.25) and body height (−0.22). On the other hand, there were correlations between the RT CHORT and standing long jump, 30 m sprint, body weight and age. The highest correlation coefficient for the standing long jump is between HECOR test, which has the negative direction. The low correlation for MT was seen only in SPANT test between the standing long jump, 30 m sprint and age. For the rest of MT, there was no correlation between the mentioned variables. It is worth noting that there was a correlation between the 30 m sprint and the majority psychomotor tests, while there was correlation only between MT SIRT, MT CHORT and MT HECOR. A similar situation was between the age and psychomotor tests, where the same tests, in the standing long jump, did not show any correlation between the variables.

The next stage of the analysis was a particular assessment of the influence of age, physical fitness and anthropometric parametres on cognitive abilities. Table 5 presents the models with errors of fitting calculated for the reaction time, motor time and the number of errors performed during computer tests. The analysis showed that the most accurate model is the model for HECOR RT, which characterized by the error NRMSE=11, while the model for SPANT MT for which the error was NRMSE=34 was the smallest fit. It is also noted that in each test the models for reaction time are much more accurate than the models for motor time. The analysis with the use of forward selection multiple regression also made it possible to determine the optimal systems of variables for individual models. The simplest model structure was observed for CHORT RT, it only takes into account age and height. The most complex structure was calculated for CHORT MT where the model takes into account age, height, body weight, BMI and both technical indicators.

## 4. Discussion

The main aim of the study was to assess cognitive abilities, motor skills and age among children and teenagers practicing football. The research showed the best level of cognitive abilities in the study was shown in the age group G4 (14–15 years old) and G5 (16 years old), where both groups had similar results. The youngest group in each test had the lowest test results which shows that with age, the level of cognitive abilities develops until around 14 years old. Multiple regression analysis shows that age has an effect on every psychomotor test except simple reaction time (SIRT). The correlation between age and the cognitive abilities of children was confirmed by Allen and Ondracek in their research [48]. The relationship between the results of psychomotor tests and age was also analyzed by Montés-Micó et al. [34]. The studies were also divided into age categories (8–9 years, 10–11 years and 12–13 years) and it was observed that with age, the times obtained in psychomotor tests are shorter. Similar conclusions were obtained during the studies conducted by Beavan et al. [40] where the reaction time of young footballers was related to age. The improvement of complex reaction time with age in young footballers was confirmed in the study of Hirose et al. [42] where the complex reaction time decreased to the age of 14.

The post-hoc analysis shows that the older age groups, the more non-statistically significant differences between the level of psychomotor abilities. Similar results were obtained in the study of Figueiredo et al. [33], where no statistically significant differences were found between psychomotor abilities in the age groups of 11–12 years and 13–14 years old. Penna et al. [41] showed that in the group of 13 years old children (13.36 ± 0.45), divided into two age groups according to the time of birth, there were no significant differences in reaction time and motor abilities. On the other hand, in the younger age group there are many statistically significant differences in the results of cognitive tests, which is also confirmed by the studies of Iida et al [44]. The authors observed that reaction time significantly improved between 6–12 years old.

In the research, the authors observed that the number of correct answers in CHORT and SPANT tests improved with age, which was also associated with the highest correlation. (Figure 1). Similar results were presented in the study made by Schumacher et al. [2], where there was also a high correlation between the age of young football players and correct answers in perceptual-cognitive tests. The best results of attention abilities during psychomotor tests were achieved in adolescence [49,50].

In the presented studies, the reaction time of individual psychomotor tests had a statistically significant correlation with the results of fitness tests. The result of standing long jump and 30 m sprint correlated most strongly with the reaction time in each psychomotor test. Similar conclusions were reached in research made by Wrotniak et al. [51] where observed that physical activity in children is positively related to psychomotor performance. Another study on young football players from Valencia Soccer Club showed that youth footballers achieved better reaction time than non-training people [34]. Multiple regression shows that physical fitness tests only affect only hand-eye coordination. The movement time for SPANT is associated with a standing long jump, while the reaction time with the number of correct answers.In another work, Singh and Amandeep [52] found that 6-week plyometric training had positive influence on a reaction speed.

This study also showed the correlation between the level of special fitness and the results of cognitive abilities tests. The player’s technique in a 20 m shuttle running test with and without the ball showed only one weak negative correlation between the correct choices in SPANT test. The second test determining the player’s technique, i.e., slalom with the ball and without it showed no relation to the psychomotor tests. The analysis showed that indicator of technique 1 (shuttle run test) has got influence on the number of correct responses for both, complex reaction time and eye-hand coordination. The indicator of technique 2 (slalom run) has got an effect on each psychomotor test except SPANT. In the studies also made of other authors, the analyses of the correlation between the level of technology and cognitive abilities were not found. In the work of Millic et al. [53] the correlation between the player’s experience and the time of a simple and complex reaction was shown. In this work, it was observed that more experienced players had a shorter reaction time. Huijgen et al. [39], confirmed that young footballers from teams playing at the highest level of the competition are characterized by better cognitive abilities than players playing at a lower level. In the studies carried in youth academy of the Dutch League shows that boys in the talent development program of performance react quicker to situations and have a better mental state than boys training in amateur clubs [38].

While analyzing the correlation between BMI index and the level of cognitive abilities, only a low negative correlation was noted during simple reaction time and reaction time test of hand-eye coordination. The other results of psychomotor abilities tests did not show any correlation with BMI. While multiple regression analysis showed the effect of body weight and BMI on SIRT RT and MT, CHORT MT and HECOR RT and MT. However, for SPANT RT and MT the model showed a relationship only with body weight. The results mentioned above were also proved in the study made by Mohammad et al. [45] where there was no correlation between the fat tissue and reaction time either. The body height is a component of the model of all psychomotor skills, except for motor time in two-dimensional hand-eye coordination (SPANT MT). Tonneesen et al. [54] showed no correlation between the body height and the reaction time in their study.

The limitations of the study were related to the research group. Only the data of football players without the control group was analyzed in the study. A control group of non-athletes helped to contextualize the results of the study and to make a statement about the relationship between cognitive abilities, fitness levels and age for footballers. In the future the studies can be extended to the comparison of youth training football with a non-training group. The other scientists have shown that people training various sports are characterized by a faster simple reaction, which shows that through sports training we are able to improve this ability [11,15,18,21,28,55,56,57,58,59]. Ando et al. [32] confirmed that thesis during their research, showing that players who train football have faster reaction time than those who do not train. Cognitive abilities can also be compared with other disciplines. Aksoy et al. [31] while analyzing the assessment of reaction time in the group of young football players, volleyball players and wrestlers, observed that young football players had the best reaction time.

## Figures and Tables

**Table 1:** Characteristic of participants.

Age	N	(%)	Age	Body Height (cm)	Body Weight (kg)	BMI
**Total**	258	100	12.1±2.03	154.65±14.75	44.38±12.83	18.21±2.5
**G1**	28	10.9	8.7±0.5	134.4±5.68	30±5.07	16.37±2.11
**G2**	71	27.6	10.6±0.5	146.48±9.06	37.6±7.56	17.38±1.98
**G3**	105	40.7	12.4±0.5	155.56±9.38	45.04±9.67	18.48±2.65
**G4**	32	12.4	14.7±0.5	173.01±8.47	58.65±10.67	19.46±2.11
**G5**	22	8.6	16.0±0.0	174.19±11.27	61.55±10.55	20.19±1.57

Age category: G1–8–9 years, G2–10–11 years, G3–12–13 years, G4–14–15 years, G5–16 years.

**Table 2:** Numeral characteristics of psychomotor abilities of football players.

Variable	Total	G1	G2	G3	G4	G5	*p*
	N = 258	N = 28	N = 71	N = 105	N = 32	N = 22	
SIRT							
RT [ms]	382.9±52.5	412.9±40.7	394.3±53.9	371.3±56	373.3±34.9	377.4±46.1	0.0002 *
MT [ms]	189.5±49.1	198.9±44	188.9±48.8	177±43.4	198.3±47.1	225.8±63.8	0.001 *
CHORT							
RT [ms]	741.4±86.5	792.6±78.4	765.6±89.7	726.3±80.5	712.6±58.3	711.4±104.3	0.0001*
MT [ms]	219±66	271.6±99.5	224.9±61.4	199.9±53.2	213.3±52.8	244.1±59.8	0.0001 *
c.r. [%]	83.1±14	69.6±16.3	79.7±13.8	85.8±12.1	91.5±7.1	86.1±13.9	0.0001 *
HECOR							
RT [ms]	454.6±54,5	507.1±49.4	465.1±54.7	441.5±49.5	437.6±40.1	441.4±56.8	0.0001 *
MT [ms]	237.1±52	269.5±55.8	242.3±55.8	220.5±43.8	238.6±54.2	258.6±53.6	0.0002 *
SPANT							
RT [ms]	693.5±122.4	759.6±178.3	726±112.7	681.2±111.3	641.9±91.4	637.1±93.9	0.0001 *
MT [ms]	300.8±106.6	369.7±153.8	337.8±118.2	272.8±85.2	257.6±58.6	289.1±55.5	0.0002 *
c.r. [%]	75.4±23.1	51.3±27.1	67.8±22.9	79.9±19.1	88.4±12.4	90±15.1	0.0001 *

SIRT–Simple Reaction Time, CHORT–Choice Reaction Time, HECOR–Hand-Eye Coordination Test. SPANT–Spatial Anticipation Test, RT–reaction time, MT–movement time, c.r.–correct responses. Age category: G1–8–9 years, G2–10–11 years, G3–12–13 years, G4–14–15 years, G5–16 years, * statistical significance.

**Table 3:** Post-hoc analysis.

	SIRT		CHORT			HECOR		SPANT		
	RT	MT	RT	MT	c.r.	RT	MT	RT	MT	c.r.
G1 vs. G2	*	NS	NS	*	**	***	*	*	NS	**
G1 vs. G3	***	*	***	***	***	***	***	***	***	***
G1 vs. G4	***	NS	***	*	***	***	*	***	***	***
G1 vs. G5	**	NS	**	NS	***	***	NS	***	*	***
G2 vs. G3	*	NS	***	**	**	**	*	**	***	***
G2 vs. G4	NS	NS	***	NS	***	*	NS	***	***	***
G2 vs. G5	NS	*	*	NS	*	NS	NS	***	NS	***
G3 vs. G4	NS	*	NS	NS	*	NS	NS	NS	NS	*
G3 vs. G5	NS	*	NS	NS	*	NS	NS	NS	NS	*
G4 vs. G5	NS	NS	NS	NS	NS	NS	NS	NS	*	NS

Age category: G1–8–9 years, G2–10–11 years, G3–12–13 years, G4–14–15 years, G5–16 years. * < 0.05, ** < 0.01, *** < 0.001, NS–non statistical.

**Table 4:** Numeral characteristics of physicial fitness test of football players.

	Standing Long Jump (cm)	Sprint 30 m (s)	Technique Indicator 1	Technique Indicator 2
Total	177.96±30.56	5.26±0.48	4.27±1.39	3.52±1.52
G1	147.22±17.66	5.81±0.33	5.1±1.6	4.69±2.05
G2	165.35±21.05	5.48±0.36	4.55±1.49	3.56±1.24
G3	180.64±21.45	5.24±0.36	4.07±1.24	3.42±1.66
G4	202.56±44.12	4.8±0.34	3.66±1.12	2.97±0.71
G5	209.86±26.78	4.7±0.41	4.24±1.25	3.19±0.82

Age category: G1–8–9 years, G2–10–11 years, G3–12–13 years, G4–14–15 years, G5–16 years.

**Table 5:** Forward selection multiple regression models.

Variable	RMSE	NRMSE	Regression Model
SIRT
RT	50.3 ms	13	y=934.3−416.8·BH+4.8·BW−6.8·BMI
MT	49.7 ms	26	y=503.7−244.1·BH+4.6·BW−8.5·BMI+3.6·T2
CHORT
RT	83.9 ms	11	y=983.2−5·AGE−119.9·BH
MT	67.1 ms	31	y=662.2−4.8·AGE−307.1·BH+5.4·BW−10.3·BMI+6.6·T1+3.5·T2
cr	13.2%	16	y=38.9+1.2·AGE+23.3·BH−1.3·T1
HECOR
RT	50.9 ms	11	y=991−6.3·AGE−359.8·BH+4.7·BW−6.8·BMI+2.5·T2
MT	52.7 ms	22	y=764.2−3·AGE−363.2·BH+6.4·BW−12.5·BMI+4.6·T2
SPANT
RT	118.7 ms	17	y=1250.5−13.9·AGE−299.3·BH+2.3·BW−8.9·T2
MT	103 ms	34	y=470.3−15.5·AGE+4.7·BMI−0.4·SLJ
cr	20.4%	27	y=−66.5+4.3·AGE+48.4·BH−1·BMI+7.9·S30−1.8·T1

BH–Body height, BW–Body weight, T1–Shuttle running. T2–Slalom run, SLJ–Standing long jump, S30–Sprint 30 m.

## Data Availability

We wish to thank all volunteer participating young football players.

## Sample Availability

Samples of the compounds are available from the authors.
